# How social opinion influences syntactic processing—An investigation using virtual reality

**DOI:** 10.1371/journal.pone.0174405

**Published:** 2017-04-06

**Authors:** Evelien Heyselaar, Peter Hagoort, Katrien Segaert

**Affiliations:** 1 Neurobiology of Language Department, Max Planck Institute for Psycholinguistics, Nijmegen, The Netherlands; 2 Donders Institute for Brain, Cognition and Behaviour, Nijmegen, The Netherlands; 3 School of Psychology, University of Birmingham, Birmingham, United Kingdom; Max Planck Institute for Human Cognitive and Brain Sciences, GERMANY

## Abstract

The extent to which you adapt your grammatical choices to match that of your interlocutor’s (structural priming) can be influenced by the social opinion you have of your interlocutor. However, the direction and reliability of this effect is unclear as different studies have reported seemingly contradictory results. We have operationalized social perception as the ratings of strangeness for different avatars in a virtual reality study. The use of avatars ensured maximal control over the interlocutor’s behaviour and a clear dimension along which to manipulate social perceptions toward this interlocutor. Our results suggest an inverted U-shaped curve in structural priming magnitude for passives as a function of strangeness: the participants showed the largest priming effects for the intermediately strange, with a decrease when interacting with the least- or most-strange avatars. The relationship between social perception and priming magnitude may thus be non-linear. There seems to be a 'happy medium' in strangeness, evoking the largest priming effect. We did not find a significant interaction of priming magnitude with any social perception.

## Introduction

Mimicry is one of those psychological behaviours that everyone has witnessed and/or produced themselves: at one point or another we have attempted to directly copy someone else's utterance (perhaps with a mocking tone), tried to mirror someone's movements, or attempted to put on an accent that wasn't our own. However, besides the conscious mimicry we engage in (or observe), there is also a wide range of mimicking behaviour we engage in without being aware of it. The most well-known example of this is of participants mimicking each other's foot-tapping behaviour [1]. This "chameleon effect" has also been observed in a wide range of behaviours, from performance on an intelligence test to walking speed [2–4]. What makes this behaviour so interesting is that these participants were not aware they were changing their behaviour and were primed by subtle manipulations such as using key terms hidden in scrambled sentences or questionnaires. Studies investigating this effect have also shown that not everyone mimics to the same extent [5]. This variability can be attributed to, among other things, characteristics of the social interaction, such as the likability of the person one interacts with. Indeed, there have been studies suggesting that participants are more likely to mimic people they like, compared to those they dislike [6].

Although there has been some controversy on whether these results are replicable [7,8], these studies have inspired others to investigate whether this mimicking behaviour extends to other domains. Indeed, this behavioural priming effect can also be observed in the language domain: participants adapt their speech rate and accent [9,10], even lexical [11], and grammatical [12–14] preferences, to name but a few, to match their interlocutor. Studies have shown that social factors can also influence the rate of language mimicry [15] however these studies have only looked at the influence of social factors on what can best be described as superficial language traits: body language/posture [16], speech rate [17], vocal intensity [18], etc. For higher level language change, such as lexical choice and syntactic structure, which represent changes in actual language processing, it is established that there is a priming effect, but less research has investigated whether there is an influence of social factors.

As language is a social behaviour, the suggestion that the opinion one has of their interlocutor could influence one's language processing, also at the core levels of semantic and syntactic processing, is not surprising. Although not focusing on mimicry, there have been several empirical studies that have illustrated that language processing, at the level of semantics as well as grammar, can be affected by social information. For example, participants show different brain patterns when statements were incongruent with inferred speaker characteristics [19,20], or slowed reaction times when the grammatical gender of a sentence does not match the gender of the speaker [21,22]. These studies suggest that when processing language, we already take speaker characteristics into account, and hence the assumption that something as abstract as social opinion could also have an influence is not as far-fetched.

For the rest of this paper we will focus exclusively on the adaptation of syntactic structure choices to an interlocutor’s, and refer to it as structural adaptation or structural priming.

In 2005, Balcetis and Dale [23] set out to answer whether structural adaptation is influenced by social factors by manipulating the opinion participants had of their interlocutor. Participants and confederates were invited to complete a picture description task together, with the aim of measuring how often the participant would mimic the grammatical structures used by the confederate. Before the experimental session began, the participant and confederate first completed a questionnaire so that they could “get to know each other” (p. 185). The manipulation was such that the confederate would answer the questions in either a mean or nice way. The questionnaires were exchanged such that each could read the answers of the other and, at the request of the experimenter, the participant and confederate stated what they thought of the other. To ensure that the “mean” confederate was indeed regarded as such, when in the mean condition, the confederate would answer that they would not be friends with the participant, whereas the “nice” confederate (although both styles of confederate were played by the same person) would say that they could be friends and thought the participant was “ambitious and exciting” (p. 186). They would then conduct the picture description task together.

This study showed that participants were *more* likely to mimic the structure of the sentences produced by the *nice* confederate compared to the mean confederate for 3 out of 4 structures measured. Prepositional-object (PO), active, and passive sentences all showed a significant increase in the magnitude of the priming effect with the nice confederate compared to the mean one. Only double-object (DO) structures showed a (non-significant, *p* = .13) opposite correlation. Interestingly, in Experiment 2 of the Balcetis and Dale study, the opinion of the confederate was no longer manipulated by how they profiled themselves, but rather by how they interacted with the experimenter. The mean confederate in this experiment would complain when the experimenter pretended to have difficulty in setting up the experiment, while the nice confederate would be patient and understanding. Even with this more subtle manipulation, an independent group of participants rated the confederate's behaviour as significantly different. In this study, the participant mimicked *more* sentences with the *mean* confederate compared to the nice confederate, even for DO structures. These results highlight not only how even small changes to the perception of the confederate can largely influence the results but also puts into question the stability of the results in general. Is there a consistent and stable influence of social opinion on language mimicry?

In 2014, Weatherholtz and colleagues [24] conducted a similar experiment, but instead of the binomial “mean” versus “nice” confederate they had participants complete a survey which measured how similar the participants found themselves to their partner, giving a wider spread of ratings. The experiment was conducted online, with participants first hearing a political diatribe after which they were asked to describe 10 simple line drawings. The authors manipulated the political ideology of the diatribe (political ideology of the participant was also measured in the survey) as well as the accent of the speaker. In this study, Weatherholtz and colleagues showed a *decrease* in PO priming with *increasing* similarity scores, and a non-significant (*p* = .3) increase in DO priming with increasing similarity scores. Assuming that we like people more if we find ourselves similar to that person, we can consider the results of the Weatherholtz study to be contradictory to the results of Experiment 1 of Balcetis and Dale, yet in line with Balcetis and Dale's Experiment 2. This calls into question what Balcetis and Dale, and Weatherholtz and colleagues were actually measuring. The Weatherholtz study did not have their participants take turns with their confederate in describing pictures; instead they were given ten pictures to describe in a separate experimental phase. As their results are more in line with Balcetis and Dale's Experiment 2, in which there was also no direct manipulation between the confederate and the participant, perhaps these results are more in line with mimicry in a monologue context (i.e. without the direct influence of the interaction partner).

Regardless, both studies clearly provide evidence that social factors *can* influence language mimicry at the level of syntactic processing or structure choices, however, the direction of this effect is not clear. Social factors are inherently noisy due to the personal nature of this type of measurement. For example, a person that you greatly dislike will be the best and most-trusted friend of someone else (the origin of the English saying “there is a lid for every jar”). However, we believe that the manner in which the previous studies represented the interlocutor (confederate or online recording) may have also added noise to the data, potentially resulting in the conflicting results.

In language priming studies, it is important that the participants are equally exposed to all grammatical structures that the experiment is attempting to prime. Therefore, when conducting a priming study in a dialogue context, a scripted partner (whether it be a confederate or a recording) is necessary to ensure enough exposure to each eligible structure. However, the use of a confederate has recently been scrutinized (see [25] for a review) in terms of the potential for artificially produced signals that may influence the behaviour exhibited by the participant. In most priming studies this is not necessarily an issue, as all that is necessary is to observe an effect of structural priming on the participant’s syntactic choices. However, when using a confederate to manipulate the social factors towards that confederate, even uncontrolled minute changes in the confederate’s behaviour could influence the participant’s behaviour, as was illustrated in the diverging results between Experiment 1 and Experiment 2 in the Balcetis and Dale study, for example.

Weatherholtz and colleagues addressed these issues by replacing a confederate with a recording. In addition to controlling for minute changes in behaviour between subjects, this also changed the social dynamic as the participants were not directly interacting with the confederate, just listening to a recording. Even the picture description task was just the participant describing pictures; there was no retort from a confederate. This made the manipulation subtler but also potentially changed the way the manipulation affected language production, as this was not production within the social context created. Additionally, in terms of linguistic priming, a recent study by Bergmann and colleagues has shown that participants adapt their language behaviour significantly *less* when presented with only a voice recording of their partner, as opposed to a voice recording and accompanying video [26]. This study illustrated that when participants were interacting with a computer without a video of their partner, the priming magnitude was significantly different depending on whether the participant believed their partner to be a computer program (more priming) or a human (less priming) in another room. However, once a video of their partner was added, linguistic priming magnitude (they tested both lexical and structural priming) was no different between belief conditions. This leads us to our approach in addressing the question of the relationship between social factors and language mimicry: by use of a computer with video.

In a recent study, we showed that participants adapt their grammatical structures to the same extent when interacting with a program in virtual reality (hereafter “avatar”) as when they are conducting the same task in the physical world with a confederate [27]. In this study, participants completed a picture description task together with an avatar or a human confederate. The target pictures could be described using an active or a passive sentence, and our results showed an increase in the use of passive structures following a passive prime that was of the same magnitude regardless whether the partner was the avatar and the human confederate. Crucially, this priming magnitude disappeared when participants interacted with a computer-like avatar: this avatar did not have any facial expressions, did not look at the participant and had a computerized voice. We interpreted these results as indicating that if the computer partner is human-like enough, then participants will exhibit the same behaviour towards it as they would a human partner, a claim supported in the human-computer literature [28,29].

These results, together with those of Bergmann and colleagues mentioned above, suggest that avatars in virtual reality could be a viable replacement for confederates, particularly in investigating the role of social factors. Indeed, this idea is not new [30,31] and although the effect of social factors influencing language production has not been investigated using computers, a large repertoire of studies from the Clifford Nass lab in Stanford as shown that participants do attribute social factors to machines(see for review [32]), and that these participants believe that the computer will reciprocate these feelings. Indeed, they conducted several tests on reciprocity: if a computer is particularly helpful (in terms of search ability; [33]) or discloses sensitive information (“I rarely get used to my full potential. What is your biggest disappointment in life?”; [34]), participants respond by being helpful towards the computer (in helping the computer with programming a task) or disclosing sensitive information, compared to switching to a different (yet identical) computer. These studies have suggested that participants exhibit social characteristics towards computers and therefore it is not unfair to predict that the same will occur for language mimicry.

Hence the aim of this paper is two-fold: 1) we aim to show that social factors can influence structural priming when interacting with avatars and 2) we aim to determine *how* social factors can influence structural priming, with the claim that this should reflect the directionality in human-human studies as well. In Experiment 1 we detail how we picked our three avatars. Previous studies have shown that participants prime less with computer partners (avatars included) when they are perceived as being less human [27,35]. We will therefore create and test six avatars and pick three that are rated as least human, most human and intermediate. Of course it is imperative that the avatars are still relatively human-like; if the avatar is completely computer-like, participants don't prime at all (Experiment 2 in [27]; [35]). Three human-like avatars will then be used as partners in a within-subjects structural priming task (Experiment 2), looking at the rate of priming for active and passive structures as a function of social opinion. We base our manipulation on the methodology employed by Weatherholtz and colleagues: a subtle manipulation by changing the behavioural features of the avatar, not an explicit manipulation as was done by Balcetis and Dale. We believe that this will more realistically reflect how social factors could influence language production in everyday life. Therefore, we will use the same survey used by Weatherholtz and colleagues to determine interpersonal similarity to each avatar as the independent variable against which to correlate priming magnitude.

This study will therefore answer how social factors influence language adaptation. We predict, based on the scarce previous literature, that in a social context (as in Balcetis and Dale’s Experiment 1) we should see an increase in structural priming with increased similarity/likeability of the avatar. If we can unequivocally show this effect, then language adaptation would reflect the patterns seen in social psychology for behaviour priming, suggesting a similar, non-language specific, mechanism is being used in processing syntax.

## Experiment 1

In Experiment 1, we tested six avatars with the aim to pick three that represent the least-, most- and intermediately-rated human-like avatar. Our six avatars were made out of unique combinations of different facial expressions, namely the blink rate, eyebrow movement, and smile habits. We chose to manipulate facial expressions only as, due to the nature of the structural priming task, the avatar does not move or say anything other than describing pictures. As such, we were limited in the behavioural characteristics that we could manipulate.

The participants conducted a shortened version of the structural priming task to ensure that the ratings are more relatable to Experiment 2. The participants were asked to rate the avatars on their perceived humanness, strangeness, quality of facial expressions, and quality of voice. The first three should vary, within-participants, for different avatars as a result of us manipulating their facial expressions. The quality of voice on the other hand will not be manipulated between avatars and hence will be used as a sanity check. Humanness and strangeness were included to ensure that the combinations we chose are realistic human-like expressions and do not evoke an unsettling feeling that may bias the participants structural priming behaviour as measured in Experiment 2.

## Materials and methods

### Participants

30 native Dutch speakers (13 men, M_age_: 22.5 years; SD_age_: 3.1) gave written informed consent prior to the experiment and were monetarily compensated for their participation. The study was approved by the ethics commission of the Faculty of Social Sciences at Radboud University, Nijmegen (Ethics Approval # ECG2013-1308-120).

### Materials

#### Avatars

**Fig 1:**
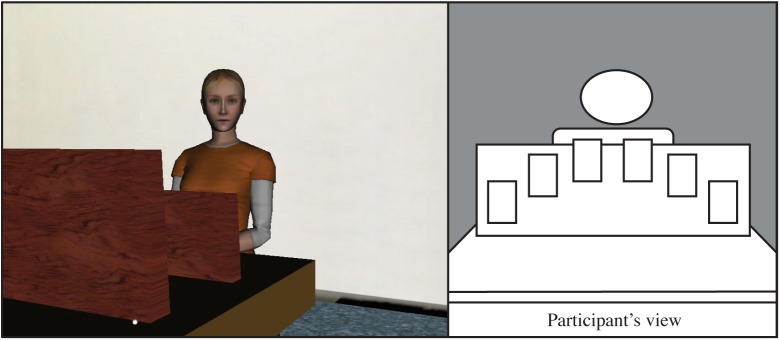
Avatar and Set-up. **A.** The exterior of the avatar was identical for all avatar partners. **B.** The experimental set-up from the view of the participant.

All avatars had the same exterior adapted from a stock avatar produced by WorldViz ("casual15_f_highpoly"; Fig 1A). All the avatars' speech was pre-recorded by the same human female and played during appropriate sections of the experiment. The avatars' appearance suggested that she was a Caucasian female in her mid-twenties, which matched the age and ethnicity of the Dutch speaker who recorded her speech.

The six facial expressions to be tested involved combinations of subtle changes in blink rate, smiling and eyebrow habits (Table 1). Blinks happened once every 1–5 seconds. For versions with dialogue-matched smiling and dialogue-matched eyebrow habits we explicitly programmed when the avatar would smile and/or raise her eyebrows, such that it would coincide with the content of her speech. For example, the avatar would raise her eyebrows when asking a question and smile when she was enthusiastic. When not speaking, she would smile once every 5–10 seconds and raise her eyebrows once every 1–5 seconds such that she would still differ from the no smile/no eyebrow version. All of these changes were extremely subtle to ensure that they can still be related to ecologically valid behavioural characteristics that one would encounter in the everyday world.

**Table 1:** Avatar Facial Expressions.

Avatar	Blink Duration^1^	Smiling Habit	Eyebrow Habit
1	No blink	No smile	No movement
2	0.5 sec (Slow)	Once every 3–5 sec	No movement
3	0.5 sec (Slow)	Constant smile	Constantly up
4	0.1 sec (Normal)	No smile	Once every 3–5 sec
5	0.1 sec (Normal)	Dialogue-matched	Once every 3–5 sec
6	0.1 sec (Normal)	Dialogue-matched	Dialogue-matched

^1^Measured from the beginning of the closing movement to when the eye is fully open again.

#### Virtual environment

The virtual environment (VE) was a stock environment produced by WorldViz ("room.wrl") adapted to include a table with a wooden divider. We chose to have the cards displayed at the top of the divider so that the participants could see the cards while facing forward (Fig 1B). This was done due to the weight of the head-mounted display (HMD), which would cause an uncomfortable strain on the back of the participants' heads when they face down. Having the participants face forward throughout the entire experiment distributes this weight more comfortably.

The experiment was programmed and run using WorldViz's Vizard software. Participants wore an NVIS nVisor SX60 HMD, which presented the VE at 1280 x 1024 resolution with a 60-degree monocular field of view. Mounted on the HMD was a set of 8 reflective markers linked to a passive infrared DTrack 2 motion tracking system from ART Tracking, the data from which was used to update the participant's viewpoint as she moved her head. It is known that this type of headset can cause dizziness and nausea due to the exclusion of the participant's nose in the field of view [36]. However, as each experimental block was quite short (~5 minutes), none of our participants reported feeling any nausea.

Additionally, a single reflective marker was taped onto the index finger of the participant's dominant hand. This marker was rendered as a white ball in the VE, such that participants knew the position of their finger at all times. Sounds in the VE, including the voice of the avatars, were rendered with a 24-channel WorldViz Ambisonic Auralizer System.

### Procedure and task

The participants were informed that they would be rating six different avatars. Exposure to each avatar started with the avatar giving a short introductory speech, followed by a card matching game. The order of the avatars was randomized and counterbalanced across participants, such that each participant interacted with all six types of avatar in all possible order combinations.

The card game is identical to the one used in Experiment 2 (for more details see *Materials and methods* of Experiment 2). But briefly: the participant and the avatar would alternate in describing picture cards to each other. If the listener saw the card described by their partner as one of the cards in their spread they would select it, causing it to be automatically replaced by a novel card. The listener would then become the speaker and pick a card to describe. This continued until 10 cards were described, after which the headset was removed and participants were asked to fill out a pen-and-paper questionnaire. We favoured a pen-and-paper questionnaire instead of having the avatar ask the questions directly as previous research has shown that if the participant evaluates the avatar in the presence of said avatar, they rate them more favourably [37]. The task was too short, however, to measure stable priming tendencies [27].

The questionnaire consisted of four 6-point Likert-scale questions asking to rate the avatar on perceived humanness, strangeness, quality of their facial expressions, and quality of their voice in relation to the other avatars. The scale was such that 1 referred to least human/least strange/lowest quality, whereas 6 referred to most human/most strange/highest quality. The latter is a sanity check as the voice is the same for all six avatars. After each avatar, the participants could change their ratings for previously viewed avatars.

## Results and conclusion

**Fig 2:**
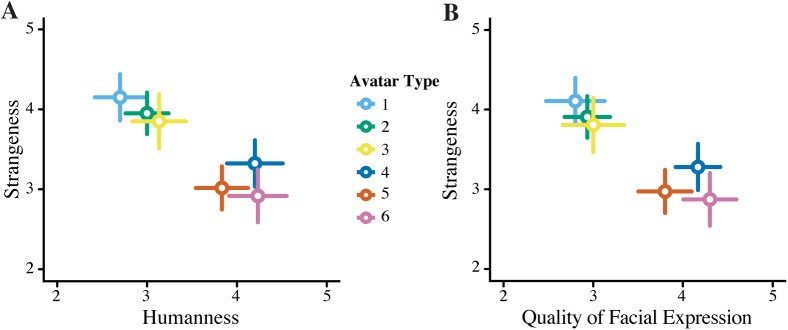
Rating of Avatar Versions for Experiment 1. **A.** Correlation between the strangeness and humanness ratings, and **B.** Correlation between the strangeness and quality of facial expressions for the six avatars. Error bars represent standard error.

We found a significant effect of avatar versions on the rating of humanness (F = 4.970, *p* < .0001), strangeness (F = 3.065, *p* = .01; Fig 2), and quality of facial expression (F = 5.097, *p* < .001). The voice ratings were not found to be significantly different between avatar versions (F = 1.418, *p* = .220), which functions as a sanity check as the voice was exactly the same for each avatar.

A *post hoc* Tukey HSD test revealed that avatars with eyebrow movement (Avatars 3–6) were rated significantly more human than avatars without eyebrow movement (Avatars 1 and 2, *p* < .05), whereas smiling habits made no significant impact on humanness rating (Avatars 1 and 4 vs. Avatars 2,3,5 and 6). This result is consistent with previous literature showing that the eyes are used to determine agency in humanoid objects [38]. Those experiments used inanimate photographs as their main stimuli, and therefore it is interesting that we were able to replicate those effects here with animated, interactive beings.

The aim of this experiment was to pick three avatars that are the least human, most human, and an intermediate without being rated as overly strange. As all six were rated under 50% for strangeness, we chose the avatars with the lowest humanness rating (Avatar 1), highest humanness rating (Avatar 6), and one intermediate (Avatar 5).

## Experiment 2

In this experiment, a new set of participants were invited to complete the full structural priming experiment with the three avatars that we chose based on the ratings obtained in Experiment 1. Each participant interacted with all three avatars. The within-subjects manipulation allows us to more accurately capture how changes in opinion can influence structural priming behaviour. As social opinion is abstract and personal such that an avatar that one participant rates highly, another participant might rate less highly, our results could become less coherent if run as a between-subjects design. By having one participant interact with all three, it will be easier to investigate how a single participant’s behaviour changes as they interact with different avatars. For each avatar, participants were again asked to rate the avatar on humanness and strangeness, as well as complete a questionnaire evaluating the interpersonal distance with the avatar, based on the questionnaire used in the Weatherholtz and colleaguse paper. The aim of the current experiment is to determine whether these ratings correlate to the magnitude of the structural priming effect, to show how social perception can influence priming behaviour.

## Materials and methods

### Participants

66 native Dutch speakers (24 men; M_age_: 21.08 years, SD_age_: 2.179) who had not participated in Experiment 1 were invited to complete a structural priming experiment with each of the three avatars. All participants gave written informed consent and were monetarily compensated for their participation.

### Statistical power

Statistical power was calculated using simulated priming data produced by the sim.glmm package [39] in R [40]. For our simulated data set we assumed 15 repetitions per prime type (active, passive, baseline; see below). We assumed a 10% passive priming effect (10% more passives produced following a passive prime compared to baseline), which is the order of magnitude commonly seen in the literature for priming studies using a picture description task, no lexical overlap, comprehension to production (participant primed by recording) as well as comprehension to production (participant primed by confederate), as well as production to production (participant primed by self) [27,41]. We simulated a maximum difference of 6% [27] between one end of the social perception scale and the other. With 66 participants, this would give our study a power of 0.957 (0.9414 0.9685; 95% confidence interval).

### Materials

#### Avatar

Avatar 1, 5, and 6 (see Experiment 1) were used to represent the least, intermediate, and most human avatar, respectively.

#### Virtual environment

The Virtual Environment is the same as the one described in Experiment 1. Although the current experiment is longer than the one in Experiment 1, none of the participants reported any feeling of nausea.

#### Stimulus pictures

The pictures used in this task have been used previously [41]. Our stimulus pictures depicted 40 transitive events such as *kissing*, *helping* or *strangling* with the agent and patient of this action. Each event was depicted by a grey scale photo containing either one pair of adults or one pair of children. There was one male and one female actor in each picture and each event was depicted with each of the two actors serving as the agent, creating four possible combinations for each event (160 transitive pictures in total). The position of the agent (left or right) was pseudorandomized such that across participants a certain card would be presented with the agent on the left and the right equally. These pictures were used to elicit transitive sentences; for each picture speakers can either produce an active transitive sentence (e.g. *the woman kisses the man*) or a passive transitive sentence (e.g. *the man is kissed by the woman*).

Filler pictures were used to elicit intransitive sentences. These fillers depicted events such as *running*, *singing* or *bowing* using one actor. The actor could be any of the actors used in the transitive stimulus pictures. There were 80 filler cards for the program to choose from.

Each card consisted of one stimulus picture with the relevant verb printed underneath.

#### Questionnaire

After an interaction with each avatar, participants completed two questionnaires. The first is an Avatar Evaluation questionnaire identical to the one used in Experiment 1. For this questionnaire participants were asked to rate the avatars on a 6-point Likert scale on humanness, strangeness, quality of facial expression, and quality of voice in relation to the other avatars. The scale was such that 1 referred to least human/least strange/lowest quality, whereas 6 referred to most human/most strange/highest quality. The second was 7 questions relating to their social opinion of the avatar (adapted from [24,27]; hereafter *Interpersonal Distance Questionnaire*). These questions were phrased as statements (see Table 2 for a complete list) and the participants indicated the extent to which they agreed with each statement on a 6-point Likert scale (6 = *I absolutely agree*, 1 = *I do not agree at all*).

**Table 2:** Factor loadings for the Interpersonal Distance Questionnaire. Loadings greater than |0.4| are in bold as these items contribute most to the meaning of a factor. Loadings less than |0.1| are omitted for clarity.

	Factor 1	Factor 2
	Interpersonal Similarity	Shyness
I could be friends with the avatar	**0.75**	-0.19
The avatar is similar to me	**0.67**	0.12
The avatar appeared generous	**0.51**	-0.32
The avatar appeared intelligent	**0.56**	
The avatar appeared selfish	**0.51**	-0.13
The avatar appeared shy	-0.20	**0.92**
The avatar appeared enthusiastic		-0.22
Variance Explained	0.27	0.15

### Task and design

All participants completed a structural priming task in the VR with each avatar. The experiment was split into three blocks: each block included the syntactic priming task with an avatar plus the Avatar Evaluation and Interpersonal Distance Questionnaire which were given after the priming task was complete. After the participant had completed all blocks, they were presented a debrief form (see below). The order of the blocks was randomized and counterbalanced across participants, such that each participant interacted with all three types of avatar in all possible order combinations.

The task is adapted from a similar structural priming task done in VR [27]. The participants were instructed to describe cards alternately with the avatar. Each block consisted of 150 trials (75 prime-target pairs) randomly picked from the database of 240 cards. At the start of each block, the participant was presented with six cards, with the belief that the avatar had her own spread of six cards behind the divider. The participants were instructed to choose one card per turn to describe and to describe it using a single concise sentence (e.g., *the man kisses the woman*). If either conversation partner had the card that was described, the card was automatically removed from the deck after the listener selected it. In truth, the avatar had an identical deck to the participant and therefore always had the card, however, would only indicate so to the participants if the card description met the necessary criteria (see *Analysis*). The six-card design was used as it creates the illusion that the avatar can understand the participant (they were told that the avatar works with a speech-detection system). This will ensure that the participant is priming with each avatar as an individual and not with the program (for a discussion on this see [31]. All participants were asked during the debrief whether they believed in this manipulation; if not, they were not included in the data set (5 out of 71 participants were discarded *a priori* for this reason).

The avatar was programmed to randomly pick one of the participant’s cards to describe; thereby ensuring that the participant always had the card described to them. The avatar was programmed to use 50% passive descriptions, 50% active descriptions. At the beginning of the block, the avatar would always go first, thereby serving as the prime for the participants’ subsequent target descriptions.

There were two priming conditions: active priming trials (active prime followed by a transitive target) and passive priming trials (passive prime followed by a transitive target). There was also a baseline condition: the avatar would describe an intransitive card (thereby not using an active nor passive structure) and the participant would respond with a transitive card. This condition was used to measure the tendency for the participant to use active and passive structures without being primed.

However, as the participant was free to choose a card to describe, the chance existed that the participant would describe an intransitive card as a target. These trials cannot be categorized as passive or active and as such cannot be used in the analysis. Therefore, to ensure an adequate number of trials in each condition, out of the 150 cards 2/3 of the cards were transitive and 1/3 were intransitive. *Post-hoc* analysis showed that there was an average of 15.41 (SD: 3.316), 16.72 (SD: 3.235), and 12.34 (SD: 4.292) trials in the passive priming, active priming, and baseline conditions respectively. This number of trials per condition is common in these types of structural priming studies [27,41,42]. Participants were only included if the ratio of active to passive priming trials with each avatar was not significantly different from 1.

### Analysis

#### Priming task

Responses during the structural priming task were manually coded by the experimenter as being either active or passive. An active sentence is one where the agent of the action is named first (e.g. *the woman kisses the man*) and were coded as 0; a passive sentence is one where the agent of the action is named last (e.g. *the man is kissed by the woman*) and were coded as 1. An independent coder blind to the purpose of the experiment verified that the coding of a random sample of participants was done correctly. Target responses were included in the analysis only if 1) both actors and the verb were named correctly (as a sentence naming only one of the actors does not qualify as a transitive sentence) and 2) no unnecessary information was included in the description (this constrains the participants to using either an active or passive description). We excluded 0.53% (79 out of 14851) of the target responses because they were incorrect.

The responses were analyzed using a mixed-effects logit model, using the glmer function of the lme4 package (versions 1.1–4 [43] in R [40]). The dependent measure was a binary variable coding whether the response syntax was active (0) or passive (1). The repeated-measures nature of the data was modelled by including a per-participant and per-item random adjustment to the fixed intercept (“random intercept”), with random adjustments to the fixed effects (“random slopes”) as was supported by the data, namely, a random slope for *Prime Type* by participant. We began with a full model and then performed a step-wise “best-path” reduction procedure, removing interactions before main effects, to locate the simplest model that did not differ significantly from the full model in terms of variance explained. *Prime Type* and *Avatar* factors were dummy coded (all levels compared to a reference group; in this case baseline and avatar 0), *Gender* was sum contrast coded. Continuous predictors were centered.

#### Questionnaire

As each participant filled in the Interpersonal Distance Questionnaire thrice (once for each avatar), we conducted a multivariate exploratory factor analysis on these results. However, as this study only consists of a maximum of 198 data entries for the Interpersonal Distance Questionnaire, a value that is too low to conduct an accurate analysis, we combined our questionnaire results with that of similar studies that have used the exact same questionnaire in the same situations [27,42,44]. This boosts the total data set to 694 (Kaiser-Meyer-Olkin Measure of Sampling Adequacy: 0.78; Bartlett’s test of sphericity: χ^2^(21): 964.64, *p* < .001). Note that this dataset is only used to determine the factor loadings for each question; the factor scores included in the final analysis are only those obtained from this current study. We used parallel analysis to determine the number of factors to be returned by factor analysis. The analysis indicated that 2 factors had the greatest explanatory power for the rating data. Table 2 shows the loading values for each of the 2 factors.

## Results

### Avatar ratings

**Fig 3:**
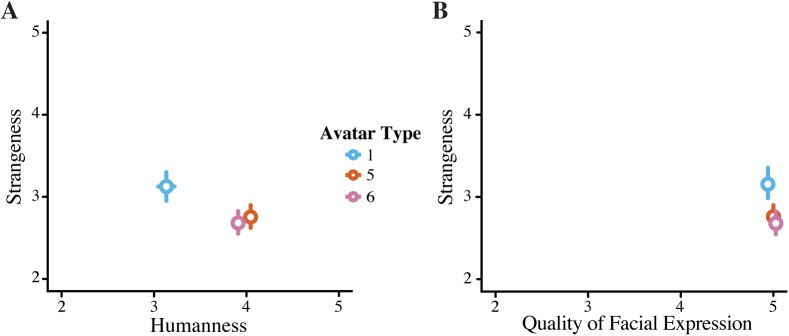
Rating of Avatar Versions for Experiment 2. **A.** Correlation between the strangeness and humanness ratings and **B.** Correlation between strangeness and quality of facial expressions for the three avatars. Error bars represent standard error.

The participants were asked to rate each avatar on humanness, strangeness, quality of facial expression, and quality of voice in relation to the other avatars to ensure that the avatars were rated the same, despite the new participant group, as they were in Experiment 1. We again found a significant effect of humanness (F = 10.668, *p* = < .001, Fig 3), with avatar 1 again being rated as the least human. However, the order of the most human and intermediate avatar was reversed in this replication: the most human avatar was now the intermediate avatar and vice versa. The relation of this new intermediate avatar to the other two is still as it was in the previous experiment: it is not significantly different from either the most or least human avatar.

We also found a significant difference in quality of facial expression (F = 12.208, *p* = < .001) and a trend in strangeness ratings (F = 2.548, *p* = .081). The ratings for voice were not significantly different between avatars (F = .174, *p* = .840), but, as in Experiment 1, this was used as a control measure as the exact same voice was used for all three avatars.

### Influences on structural priming

We assessed structural priming behaviour using a logit mixed model. We began with a full model, including avatar type and the two extracted questionnaire factors as well as interactions of all of these with *Prime Type*, and then performed a step-wise “best-path” reduction procedure, removing interactions before main effects, to locate the simplest model that did not differ significantly from the full model in terms of variance explained (Full = AIC: 4115.0, BIC: 4398.2; Best = AIC: 4077.8, BIC: 4190.6; *p* = .993). Multicollinearity was low (VIF < 2.5). *Prime Type* and *Avatar* factors were dummy coded (all levels compared to a reference group; in this case baseline and avatar 1), *Gender* was sum contrast coded. Continuous predictors were centered.

The fixed effects of the model fit for these data are summarized in Table 3.

**Table 3:** Summary of the fixed effects in the mixed logit model for the response choices based on prime structure per avatar type.

Predictor	Coefficient	*SE*	*Wald Z*	*p*	
Intercept	-3.84	0.28	-13.90	< .001	***
Gender	0.47	0.28	1.66	.100	
Active Prime	-0.66	0.24	-2.78	.001	**
Passive Prime	1.23	0.19	6.33	< .001	***
Avatar 5	0.00	0.12	0.04	.967	
Avatar 6	0.03	0.12	0.27	.786	
Cumulative Passive Proportion	0.62	0.44	8.21	< .001	***
Avatar 4 * C. Pass. Prop.	-0.75	0.53	-1.43	.154	
Avatar 4 * C. Pass. Prop	0.38	0.55	0.69	.489	

N = 8777, log-likelihood = -2022.7.

* < .05.

** < .01.

*** < .001.

The negative estimate for the intercept indicates that in the baseline condition active responses were more frequent than passive responses. This model shows a significant influence of passive primes on passive production (*p* < .001) and a significant influence of active primes on active production (*p* = .001). Therefore, we do see a robust priming effect in this experiment. There is also a significant influence of *Cumulative Passive Proportion* on passive production. This factor was calculated as the proportion of passives out of the total transitive responses produced on the target trials before the current target trial. A positive and significant *Cumulative Passive Proportion* therefore suggests that the proportion of passives previously produced positively influences the probability of producing a passive on the current target trial and is commonly used to model the learning effect of priming [27,45].

**Fig 4:**
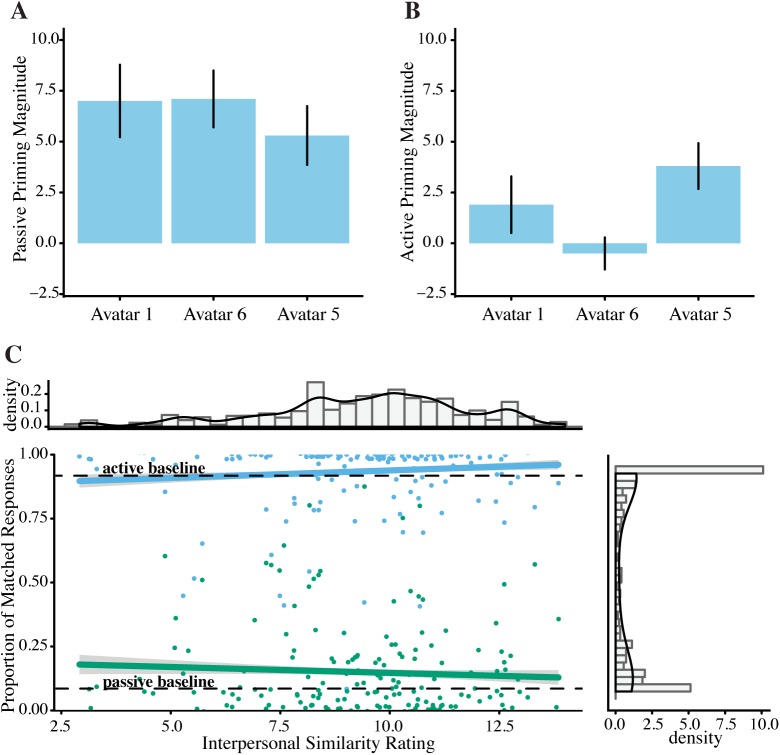
Influences on Priming Magnitude. **A.** Passive priming magnitude per avatar; although there is a significant priming effect, there was no significant difference between partner types (*p* > .05) **B.** Active priming magnitude per avatar; although there is a significant priming effect, there was no significant difference between partner types (*p* < .05). Error bars represent standard error. **C.** There was no significant effect of Interpersonal Similarity rating on priming magnitude for either structure. Error clouds represent standard error.

The model, however, does not show an effect of *Avatar* or any influence of any factor from the Interpersonal Distance Questionnaire on priming behaviour (Fig 4).

**Fig 5:**
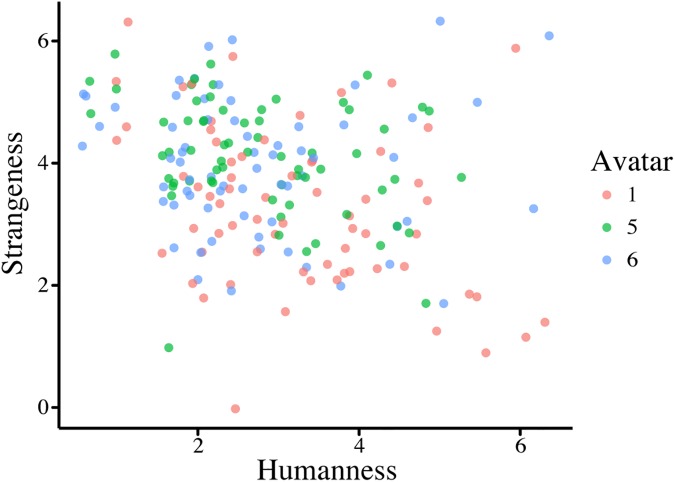
Individual ratings per Avatar. Shows that even though we used a universal manipulation, individual ratings of the avatars differ dramatically. Therefore analysis will not be done on an avatar-by-avatar basis, but as a function of each participant’s individual ratings.

Fig 4 shows that there is a trend for a difference in priming magnitude between the different avatar types, however the error bars are quite wide, even with 66 participants, which could explain the lack of statistical significance. One explanation for this is that perhaps analysing priming magnitude per avatar is not the optimal way to analyse this data. Fig 5 shows the individual ratings per avatar and it is clear that not every participant rated the avatars the same, even if the average of the ratings is significantly different (as illustrated in Fig 3). For example, some participants found avatar 1 much more human than avatar 6, even if the average of the participant group shows the reverse.

Therefore, we re-ran the mixed effects model but instead of avatar type we included each participant’s ratings for humanness, strangeness, and quality of facial expression. We began with a full model, including the two extracted questionnaire factors and the three evaluation ratings (excluding quality of voice as this was a sanity check factor) as well as three-way interactions of one questionnaire factor with one evaluative rating with *Prime Type* (e.g., Shyness*Humanness*Prime). We also included a quadratic term for humanness and strangeness *post hoc* as figures of the interaction suggested a quadratic term might better fit the data. Random slopes included *Prime Type*, *Interpersonal Similarity*, and *Strangeness (quadratic)* for Subject, and no random slopes for item. We then performed a step-wise “best-path” reduction procedure to locate the simplest model that did not differ significantly from the full model in terms of variance explained (Full = AIC: 4034.9, BIC: 4360.0; Best = AIC: 4028.6, BIC: 4247.7; *p* = .070). Multicollinearity was acceptable (VIF < 3.96). The results of the best fit model are summarized in Table 4.

**Table 4:** Summary of the fixed effects in the mixed logit model for the response choices based on prime structure per avatar ratings.

Predictor	Coefficient	*SE*	*Wald Z*	*p*	
Intercept	-3.68	0.27	-13.57	< .001	***
Active Prime	-0.77	0.25	-3.12	.002	**
Passive Prime	1.32	0.20	6.60	< .001	***
Interpersonal Similarity	0.34	0.21	1.62	.105	
Strangeness Rating (quadratic)	-0.06	0.08	-0.85	.396	
Shyness	-0.19	0.13	1.51	.131	
Cumulative Passive Proportion	3.40	0.30	11.41	< .001	***
Active Prime * Interpersonal Similarity	-0.10	0.18	-0.56	.574	
Passive Prime * Interpersonal Similarity	-0.27	0.16	-1.66	.098	
Active Prime * Strangeness Rating (quad.)	0.09	0.08	1.07	.287	
Passive Prime * Strangeness Rating (quad.)	-0.01	0.07	-0.10	.920	
Interpersonal Similarity * Strangeness (quad.)	-0.20	0.07	-3.03	.002	**
Strangeness (quad.) * Shyness	-0.15	0.05	-2.93	.003	**
Active Prime * Interpersonal Sim. * Strangeness (quad.)	0.07	0.06	1.12	.264	
Passive Prime * Interpersonal Sim. * Strangeness (quad.)	0.17	0.06	2.84	.004	**

N = 8670, log-likelihood = -1983.3.

* < .05.

** < .01.

*** < .001.

The model again shows priming effects for both active (*p* = .002) and passive (*p* < .001) structures, and again an increase in passive production over time (*p* < .001). For this model, we see influences of the ratings from the Interpersonal Distance Questionnaire and the Avatar Ratings on overall passive production, regardless of prime. This is manifested as interactions between *Interpersonal Similarity* and *Strangeness (quad*.*)* and *Strangeness (quad*.*)* and *Shyness* resulted in less passive production as these ratings increase.

In terms of effects on priming, however, we only see a significant three-way interaction between *Interpersonal Similarity*, *Strangeness*, and *Passive Prime*. This three-way interaction is hard to explain, as analysing the data by splitting the data along either *Interpersonal Similarity* or *Strangeness (quad*.*)* all provide models without a significant interaction with *Passive Prime* (*p* > .135). We will therefore investigate the contribution of *Interpersonal Similarity* and *Strangeness* on priming separately, as we had planned to before we started the analysis.

The interaction between *Passive Prime* and *Interpersonal Similarity* has been plotted above (Fig 4C), which shows a negative, non-significant influence of *Interpersonal Similarity* on passive and active priming magnitude.

**Fig 6:**
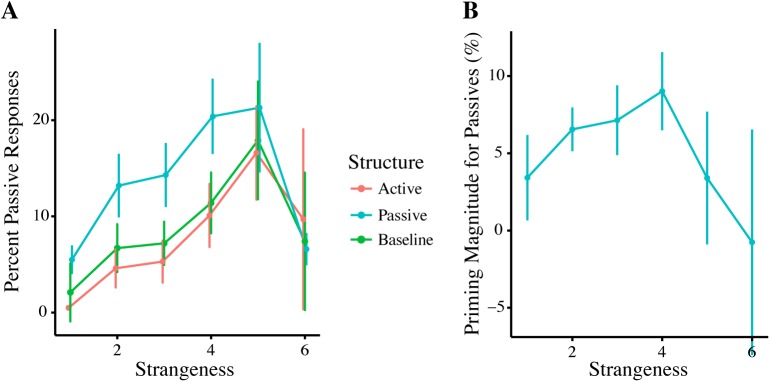
Passive Repetition per Strangeness. **A.** Percent of passive responses after each prime type **B.** Passive priming magnitude (the percent of passive responses after a passive prime after baseline correction) shows an inverted U-shaped curve as a function of *Strangeness*. Error bars reflect standard error.

Fig 6B illustrates how the magnitude of the passive priming effect changes with increasing strangeness ratings: as the strangeness rating increases, the passive priming magnitude increases as well, however, past the (roughly) midpoint of the scale, this upward trend seems to revert itself. The difference in priming strength between the midpoint and the lowest strangeness rating is roughly tripled (3.42% vs 9.02% passive priming); for the highest strangeness rating this difference is even greater (-0.76% vs 9.02%). This difference is not due to a difference in the number of data-points for each rating, as rating 2 had the most data-points (N = 79), followed by rating 3 (N = 51).

The inverted U-shaped curve suggests that at both extremes of our manipulation, participants were least likely to repeat the passive structures of the avatar, whereas in the middle of the manipulation they were most likely to repeat passive structures, an observation that has not been shown before. The active prime condition does not show this relationship and no relationship is seen in relation to the humanness rating of the avatars.

## Discussion

In this study we aimed to determine how social factors influence structural priming behaviour. As previous studies have reported conflicting conclusions, the direction and strength of influence that social factors have on the magnitude of the structural priming effect remained equivocal: do we adapt our language behaviour more or less when we communicate with people we like?

In Experiment 1 we tested 6 avatars to see how human they were, and whether our manipulation (facial expressions) did not cause the avatars to be perceived as unnaturally strange. Previous studies have shown that if the avatar is not human enough, the participants do not prime at all (Experiment 2 in [27]; [35]) and therefore it was imperative to design avatar partners that will still elicit a priming effect that is comparable to human-human interactions. Our results showed that avatars with upper-face animations (in our case, eyebrow movements) were rated as significantly more human than avatars with smiling habits or faster blink rates. These results replicated a trend seen in the agency literature, a field that studies how and why humans perceive that inanimate objects such as dolls and non-humanoid robots have thoughts and feelings of their own. Studies in this field have suggested that it all depends on the animation and presentation of the eyes and upper face of the being [38], a trend that we were able to replicate here, twice. Even though the differences in our avatars were very subtle, such as an increase in blink completion (close to open interval) from 0.1 seconds to 0.5 seconds or random smile timings versus dialogue-matched smile timings, we were still able to observe strong preferences for some avatars over others. Fortunately, no avatar was rated as uncannily strange (a rating of <3 out of 6 on the strangeness rating) and therefore we picked the least human, most human, and intermediate out of the 6 avatars tested.

In Experiment 2 we validated our assumption that our avatars were human-like enough to elicit a priming effect. We observed robust priming effects for passives (6.5% increase in passive production after a passive prime compared to baseline) and actives (1.7% increase in active production after an active prime compared to baseline). In line with the human literature, there is a stronger priming effect for passives than for actives, known as the inverse frequency effect [45–48]. We additionally observed a learning effect, such that participants were significantly more likely to produce a passive structure regardless of prime type as the experiment progressed (*p* < .001). The implicit learning nature of structural priming has been discussed before using corpus data [45], as well as observed experimentally [27,49]. Thus, we have three arguments to validate our assumption that our avatars were human-like enough to elicit a priming effect akin to those seen in the human literature, with the same characteristics: 1) we observe a robust priming effect, 2) there is an inverse preference effect, i.e. there is a larger priming effect for the less frequent structure, and 3) the priming effect is cumulative over time.

However, the aim of this study was to deduce if there is an interaction between social opinion and language behaviour, and in which direction. We did not observe a significant difference in priming magnitude for each of the three avatars, and we also did not find a significant interaction between priming magnitude and interpersonal similarity with the avatars, regardless of type. We believe the lack of an interaction between interpersonal similarity and priming, which we predicted we would find based on previous literature, could be because the questionnaire was designed for human evaluations. It included questions such as "*I could be friends with my partner*" and *"I find my partner similar to myself*" which may not translate the same when evaluating these statements about a computer. For the priming magnitude per avatar type, we believe the lack of a significant interaction is because by fitting the ratings into three conditions, we may have averaged out the individual opinions of the participants. As we are interested in how the opinion a participant has of a certain avatar influences that participant's language behaviour, it does not matter whether this avatar was previously labelled as the best or least human. For this reason, we reanalysed the data per avatar rating (humanness, strangeness, and quality of facial expression) to better represent the individual impressions each participant had.

Analysing the data per ratings indicated that as the participants found the avatars increasingly strange (as an interaction with decreasing *Interpersonal Similarity* and *Shyness*) they produced more passives, regardless of prime type. Studies have suggested that the learning part of priming is supported by surprisal [45]. Perhaps the cumulative effect of using an infrequent structure and the strangeness of the partner increased the surprisal effect such that learning increased. In our analysis we had modelled learning as *Cumulative Passive Proportion* and we did not find an interaction of this factor with *Strangeness*, which argues against this interpretation. What drives this increased passive production with increasing strangeness and decreasing values in *Interpersonal Similarity* and *Shyness* is as of yet unclear.

Our novel, and most important, finding for this study is an inverted U-shaped interaction between the strangeness rating of the avatars and passive priming. This interaction was not observed for active priming nor for any of the other avatar ratings. This significant effect was found with the first 36 participants (*p* = .0004), however, as this inverted U-shaped curve was neither expected nor predicted, we recorded an extra 30 participants. The complete 66 participant set is what we have reported here, and thus we have such already, albeit indirectly, replicated the finding at least once and are confident in the stability of these results. Our claim of this interaction is based on a three-way interaction between *Interpersonal Similarity*, *Passive Prime*, and *Strangeness* and not a direct two-way interaction. However, we found no significant results when exploring this three-way interaction via median splits of *Strangeness* and *Interpersonal Similarity* and hence we are not entirely sure where this significant effect emerges from. There was a significant correlation between *Interpersonal Similarity* and *Strangeness* which may drive the three-way interaction. However, we have only indirect evidence to support this theory and hence further studies are definitely needed to further investigate this effect.

We believe that what we labelled as 'strangeness' when interacting with an avatar interlocutor, as opposed to a human interlocutor, gauges favourability towards the conversation partner. It seems that there is a 'happy medium', in our case a strangeness rating of 4 out of 6, which elicits the highest passive priming effect compared to all the other strangeness ratings (9.02%). As the ratings diverge from this middle rating, the priming effect decreases to either 3.42% for the least strange avatar, or -0.76% for the most strange avatar. This is what has been referred to in other fields as the Goldilocks principle: something must be within an ideal range to exhibit the maximum effect. There are theories suggesting that priming is a default social behaviour and therefore only occurs if there are no top-down cues to override it [4]. It could be the case that in the middle of our manipulation gradient there are no over-ruling social cues, and therefore the participants exhibit the highest probability of passive structure repetition. It is the extremes, the left and right side of the curve, in which top-down cues override their default behaviour and therefore decrease the probability of passive structure repetition.

Although unexpected, our model suggests that there is a correlation between the interpersonal similarity and strangeness rating, such that the less strange the avatar is perceived, the higher the interpersonal similarity score (*p* = .002). This, together with previous studies showing that priming magnitude is comparable between avatar and human partners [27], suggests that our results can be compared to those seen in the human literature: perhaps the difference between the Balcetis and Dale studies, and Weatherholtz and colleagues may not be a matter of social versus asocial context but perhaps the manipulations tested different ends of this inverted U-shaped curve. Perhaps watching your partner insult a third party causes a stronger opinion than having your partner be mean to just you. Therefore, Study 2 might occur on the right side of the curve in Fig 5B, whereas Study 1 might occur on the left side, hence explaining why they observe a different interaction between social opinion and priming. Overall, we predict that an inverted U-shaped curve also underlies how social perception influences syntactic priming with human partners.

Certain studies have already suggested why participants prime less on either side of the 'happy medium'. With a very high interpersonal similarity, participants might attempt to show individuality and creativity by not mimicking their partners. In a study where heterosexual mixed-gender participant pairs were invited to complete a structural priming task the likelihood that the males repeated the syntactic structure used by the female was inversely related to the female’s level of fertility [50]. The authors explain this behaviour as the need to show creativity: if the males use “novel” syntactic structures in their responses, they exhibit their creativity and therefore their candidacy as a potential mate. On the other hand, if you interact with someone whom you don't find very similar to yourself, you simply do not adopt their behavioural tendencies. This leaves only the person in the middle, who is neither too similar nor too different, that elicits the highest priming effect.

In terms of human-computer structural priming studies, there has also been a dispute with what is expected. Previous studies by Branigan and colleagues [51]and Pearson and colleagues [52] have shown that participants exhibit a higher priming magnitude with computer partners that were perceived as being less human, whereas Beckner and colleagues [34] and Heyselaar and colleagues [27] showed the opposite effect: less/no priming with a computer partner compared to human or human-like partners. This difference can be attributed to how the manipulation was conveyed: for the Heyselaar and Beckner studies, the participant interacted directly with computer partners as the partners were either an avatar presented in virtual reality, or robots seated next to the participant. For the Branigan and Pearson studies, however, the manipulations were more subtle: the participants interacted with the computer via a chat program with no video. As in the human-human structural priming studies, this difference in manipulation could cause a difference in the placement of the interlocutors on the inverted U-shaped curve, with the Branigan and Pearson studies starting at the very left, and therefore showed an increase in priming tendency with increasing strangeness perception, whereas the Heyselaar and Beckner studies started at the peak, and therefore showed a decrease in priming tendency with increasing strangeness levels.

To summarize, we were able to observe an inverted U-shaped interaction with passive structure repetition and strangeness rating of the interlocutor, a novel observation that helps piece together previously divergent studies. By taking advantage of the flexibility, yet fine control, that VR offers, we were able to establish that there is an effect of social perception on structural priming magnitude in a way not possible using traditional methods. Moreover, our results suggest that the relationship is of a non-linear nature, although further research is needed to establish the replicability of this result. In this study, we focused on positive/negative ratings although we in no way are claiming that opinion is unidimensional. The interactions between *Interpersonal Similarity*, *Strangeness* and *Shyness* clearly support that there are higher complex relationships that influence passive production. Other features such as social goals and motivation may also play a role (although see [42]). However, our study does show that there is accumulating and convincing evidence that syntactic processing is sensitive to high-level interpersonal factors that can modulate the operation of acclaimed automatic mechanisms.
